# PKM2-driven glycolysis mediates rotenone neurotoxicity via MG-Hs in Parkinson’s disease

**DOI:** 10.1038/s41598-026-54865-7

**Published:** 2026-05-30

**Authors:** Rong Li, Jia Wen Ma, Hui Chen, Dan Mu, Lang Qu, Dan Wang, Ya Zhao

**Affiliations:** 1 Department of Ophthalmology, Affiliated Hospital of North Sichuan Medical College, Nanchong, Sichuan China; 2 Medical School of Ophthalmology and Optometry, North Sichuan Medical College, Nanchong, China; 3 School of Pharmacy, North Sichuan Medical College, Nanchong, Sichuan China; 4 Institute of Basic Medicine, North Sichuan Medical College, Nanchong, Sichuan China

**Keywords:** Parkinson’s disease, Glycolytic activation, PKM2, MG-Hs, Shikonin, Diseases, Drug discovery, Neurology, Neuroscience

## Abstract

Parkinson’s disease (PD) is a progressive neurodegenerative disorder lacking disease-modifying therapies. Rotenone (Rot) is widely used to model PD, but its neurotoxicity is not fully understood beyond mitochondrial complex I inhibition. Here, we identify a glycolytic mechanism that contributes to Rot-induced neuronal damage downstream of complex I inhibition. Our in vitro data demonstrate that Rot enhances glycolytic flux, leading to accumulation of methylglyoxal-derived hydroimidazolones (MG-Hs), which drive irreversible cellular damage. Shikonin effectively attenuates Rot-induced apoptosis by inhibiting PKM2, thereby suppressing glycolysis and reducing MG-Hs formation. In a rat model, shikonin robustly improves motor function and preserves nigrostriatal dopaminergic neurons. Collectively, our findings reveal a previously unrecognized glycolytic-mediated pathway involving PKM2-driven glycolysis and MG-Hs accumulation that contributes to rotenone neurotoxicity alongside mitochondrial dysfunction, and highlight shikonin as a promising neuroprotective agent for Parkinson’s disease intervention.

**Supplementary Information:**

The online version contains supplementary material available at 10.1038/s41598-026-54865-7.

## Introduction

Parkinson’s disease (PD) is the second most prevalent neurodegenerative disorder after Alzheimer’s disease, characterized pathologically by dopaminergic neuron degeneration in the substantia nigra pars compacta^1–3^. Clinically, PD manifests with resting tremor, autonomic dysfunction, and progressive motor impairment, substantially reducing patients’ quality of life. Current therapies are mainly symptomatic (e.g., dopamine replacement) and do not halt disease progression.

The development of representative PD models is crucial for advancing therapeutic research. Commonly employed neurotoxins for PD modeling include 6-hydroxydopamine (6-OHDA), 1-methyl-4-phenyl-1,2,3,6-tetrahydropyridine (MPTP), paraquat, and rotenone (Rot)^4^. While 6-OHDA effectively induces dopaminergic neuron loss, its inability to cross the blood-brain barrier necessitates direct central administration, limiting its capacity to recapitulate comprehensive PD pathology and early disease stages. MPTP readily crosses the blood-brain barrier and induces parkinsonian features, though it typically produces acute/subacute neurodegeneration rather than the chronic progression observed clinically. Paraquat exhibits partial PD-like effects but demonstrates poor blood-brain barrier permeability. In contrast, rotenone’s high lipophilicity enables efficient penetration of both the blood-brain barrier and cellular membranes. Notably, rotenone-induced models faithfully reproduce the behavioral phenotypes and neuropathological hallmarks of clinical PD, including progressive nigrostriatal degeneration and α-synuclein pathology, making it particularly valuable for both in vivo and in vitro PD research^5–7^.

The neurotoxic mechanisms underlying rotenone-induced PD models involve multiple pathological pathways, including α-synuclein aggregation, mitochondrial dysfunction, elevated reactive oxygen species generation, compromised antioxidant defenses, and apoptotic cell death in dopaminergic neurons^8–10^. While these effects have traditionally been attributed to rotenone’s inhibition of mitochondrial complex I, emerging evidence challenges this paradigm. A pivotal study employing Ndufs4-deficient mice, which lack a critical subunit for complex I assembly, demonstrated that rotenone retains its neurotoxic effects even in the absence of functional complex I^11^. This finding suggests that rotenone-induced dopaminergic neurodegeneration may involve mechanisms downstream of, or in addition to, complex I inhibition, leaving its precise pathogenic actions still under considerable debate.

In this study, we explore the possibility that rotenone triggers neuronal damage through glycolytic activation. Our in vitro data show that rotenone increases glycolytic flux, as evidenced by lactate production, glucose consumption, and upregulation of key glycolytic enzymes. This enhanced glycolysis is associated with accumulation of methylglyoxal‑derived hydroimidazolones (MG‑Hs)—electrophilic adducts formed by non‑enzymatic reactions between the glycolytic by‑product methylglyoxal (MG) and arginine residues, which can cause irreversible cellular damage^12–14^. Under physiological conditions, MG is efficiently detoxified by the glyoxalase system (Glo1/Glo2); when glycolytic flux is excessive or detoxification capacity is overwhelmed, MG‑Hs accumulate and promote cell death. We further identify shikonin, a natural naphthoquinone with multiple reported targets (including PKM2), as a candidate neuroprotective agent. Our results indicate that shikonin reduces rotenone‑induced apoptosis, at least in part through PKM2 inhibition, thereby suppressing glycolysis and MG‑Hs formation. Together, these findings are consistent with a complex I‑independent pathway involving glycolytic dysregulation and MG‑Hs accumulation. However, given the associative nature of the current data and the multi‑target pharmacology of shikonin, the proposed pathway should be viewed as a hypothesis‑generating model that requires further mechanistic validation.

## Materials and methods

### Cell culture

The PC12 cell line was obtained from the American Type Culture Collection (ATCC), and cultured in RPMI 1640 medium (Gibco, C11875500BT) supplemented with 10% fetal bovine serum (FBS; Biochannel, BC-SE-FBS01), 50 U/mL penicillin and 50 µg/mL streptomycin (Biochannel, BC-CE-007).

### Cell differentiation

The differentiation medium was also based on RPMI-1640 with L-glutamine, gentamicin, and amphotericin B, with 1% donor horse serum (DHS) and the addition of nerve growth factor (NGF) (Sigma-Aldrich, N2513; ) at concentrations of 100 ng/mL. The culture medium was changed every 48 h^15^.

### Cell viability assay

PC12 cells were plated in 96-well culture plates and allowed to adhere for 24 h. Following initial attachment, cells were differentiated by treatment with nerve growth factor (NGF) for three days. For experimental treatments, cells were exposed to various concentrations of rotenone (Rot, Sigma-Aldrich, R8875), 2-deoxyglucose (2-DG, Sigma-Aldrich, 25972-M), aminoguanidine (AG, Sigma-Aldrich, 396494), and methylglyoxal (MG, Sigma-Aldrich, M-0252), each dissolved in DMSO vehicle. All compounds were incubated with cells for 24 h under standard culture conditions. Cell viability was subsequently assessed using sulforhodamine B (SRB) assay, with viability values normalized to vehicle-treated control groups. Experimental data represent five replicate wells per condition.

For shikonin (Selleck, HY-N0822) pretreatment experiments, cells were first incubated with shikonin (50 nM) for 2 h. Thereafter, rotenone (200 nM) was added to the culture medium without removing shikonin, and the cells were co‑incubated for an additional 24 h. Shikonin was present throughout the entire rotenone treatment period and was not subsequently removed.

### Terminal deoxynucleotidyl transferase dUTP nick end labeling (TUNEL) staining

After drug treatment, the cells present in the supernatant were pooled with trypsinized adherent cells, centrifuged, and resuspended in PBS. Aliquots of this suspensionwere deposited on slides using a cytospin centrifuge. The cells were fixed with ice-cold 4% paraformaldehyde for 10 min and washed twice with PBS. They were then subjected to TUNEL (Yeasen, 40308ES20) according to the manufacturer’s instructions. Next, 4 0 ,6-diamidino-2-phenylindole (DAPI) (1/1000 in PBS) was added to the cells for 5 min at 25 °C. Images were obtained using a fluorescence microscope (Nikon ECLIPSE 50i).

### Assessment of apoptosis by flow cytometry

Apoptosis in PC12 cells was quantitatively evaluated through flow cytometric analysis. Following experimental treatments, cells were harvested and subjected to dual staining with FITC-conjugated annexin V and propidium iodide using a commercial apoptosis detection kit (Beyotime, C1062L), in strict accordance with the manufacturer’s protocol. Stained cell populations were then analyzed using a high-performance flow cytometer (CytoFLEX LX, Beckman Coulter) to distinguish viable, early apoptotic, and late apoptotic/necrotic cell populations based on their characteristic staining patterns.

### Metabolite quantification in culture supernatants

Extracellular lactate and glucose concentrations in cell culture supernatants were determined using commercial colorimetric assay kits (lactate, Nanjing Jiancheng, A019-2-1; glucose, Nanjing Jiancheng, F006-1-1) following the manufacturer’s standardized protocols. Lactate production and glucose consumption were assessed through specific enzymatic reactions that generate chromogenic products proportional to metabolite concentrations, with absorbance measurements performed at appropriate wavelengths for each assay. Glucose consumption was calculated by subtracting the glucose concentration in the conditioned medium from that in fresh, unused medium after normalizing to total cellular protein content.

### PKM2 knockdown

PC12 cells were transfected with PKM2 (Norway rat) siRNA-468 (5 0 -CCGCAGAGGUGGAGCUGAATT-3 0 ) (siRNA PKM2^1#^), PKM2 (Norway rat) siRNA-867 (5 0 -GCAAGAACAUCAAGAUCAUTT-3 0) (siRNA PKM2^2#^), and PKM2 (Norway rat) siRNA-640 (5 0 -GGAGAAAGGUGCUGACUACTT-.

3 0 ) (siRNA PKM2^3#^) (Obio Technology (Shanghai) Corp., Ltd., Shanghai, China) using UltraFection 3.0 (FXP135-020; Beijing 4 A Biotech Co., Ltd., Beijing, China) for 24 h, and then analyzed by Western blotting (Fig. S2A, see Fig. S7A for full immunoblots).

### Plasmid transfection

For PKM2 overexpression, PC12 cells at 60%–70% confluence were transfected with an PKM2 overexpression construct (p-DNR-LIB-Pkm2(Rat)) using UltraFection 3.0 transfection reagent (Elji Biotechnology). The DNA- lipid complex (1:2DNA- to- reagent ratio) was incubated with cells in serum- free medium for 6 h at 37 °C, followed by replacement with complete growth medium. Transfection efficiency was assessed 24 h post- transfection via Western blotting (Fig. S2C, see Fig. S7B for full immunoblots).

### Western blot analysis

Following experimental treatments, cells were lysed using RIPA lysis buffer (Cell Signaling Technologies) according to the manufacturer’s protocol. Protein concentrations were determined using Bradford reagent (Bio-Rad). Equal amounts of protein (50 µg per lane) were separated by SDS-PAGE and transferred onto polyvinylidene fluoride (PVDF) membranes. The membranes were then cut into strips based on molecular weight markers prior to blocking, allowing simultaneous detection of target proteins with different molecular weights. After blocking with 5% non-fat dry milk for 1 h at room temperature, the membrane strips were incubated overnight at 4 °C with primary antibodies against PFKFB3 (13763-1-AP, Proteintech), PKM2 (15822-1-AP, Proteintech), LDHA (19987-1-AP, Proteintech), MG-Hs (STA-011, Cell Biolabs), and PARP (#9542, Cell Signaling Technologies). β-actin (20536-1-AP, Proteintech) was used as a loading control. The strips were then incubated with appropriate horseradish peroxidase-conjugated secondary antibodies for 1 h at room temperature. Protein bands were visualized using ultra ECL reagent and imaged with a ChemiDoc MP Imaging System (Bio-Rad). Quantitative analysis was performed using ImageJ software (version 1.46r; National Institutes of Health), with target protein expression normalized to β-actin.

### Rotenone-induced Parkinson’s disease rats model

#### Animals and ethical compliance

Male Sprague-Dawley rats (SD rats) were obtained from the Animal Experiment Center of North Sichuan Medical College. Animals (15–20 weeks old) were housed under standardized conditions (12:12 h light/dark cycle; 22 ± 2 °C) with free access to food and water. All experimental procedures were approved by the Animal Experimentation Ethics Committee of North Sichuan Medical College (Approval No. 2024108) and performed in accordance with NIH guidelines (Guide for the Care and Use of Laboratory Animals, Publication No. 85 − 23, revised 1996).

#### Rotenone-induced Parkinson’s disease model establishment

Rotenone was dissolved in a minimal volume of DMSO (final concentration ≤ 0.1%) and diluted with corn oil to a working stock of 2 mg/mL. The vehicle control group received corn oil containing the same concentration of DMSO (≤ 0.1%). To minimize mortality during rotenone administration, we implemented an optimized dosing protocol based on previous methodology^16^. Rotenone was administered by subcutaneous (s.c.) injection using a graduated dosing regimen: 1.0 mg/kg for the first 5 days, followed by a 25% increase every 5 days to 1.25 mg/kg, and then to 1.5 mg/kg. The vehicle control group received equal volumes of corn oil (with 0.1% DMSO) following the same escalation schedule. All injections were given in the scruff of the neck, alternating sides, with a volume of 1 mL/kg body weight. Animals were handled and injected daily using an identical protocol to control for injection‑related stress. Animals were acclimated to handling for one week before the start of the experiment. Animals were euthanized upon exhibiting severe parkinsonian symptoms, including marked rigidity and akinesia that impaired normal feeding and grooming behaviors.

#### Drug administration

Shikonin (SK, 5 mg/kg/day; Selleck, Shanghai, China) was suspended in a vehicle consisting of 5% dimethyl sulfoxide (DMSO), 10% Tween‑80, 30% PEG300, and 55% saline. The vehicle control group received an equal volume of the same vehicle without shikonin. Both shikonin and vehicle were administered once daily by intragastric gavage for three consecutive weeks. Rotenone injection (as descry ibed above) was started on the same day as the first shikonin/vehicle administration, and shikonin treatment continued throughout the entire rotenone exposure period.

#### Behavior tests

Rotarod test. Motor function was evaluated using an accelerating rotarod apparatus according to established protocols^17^. Following a 3-day acclimatization period, animals were placed on the rotating cylinder with gradual speed progression from 4 to 30 rpm over a 2-minute interval. Each trial was terminated when either: (1) the animal fell from the apparatus, or (2) maintained grip while failing to ambulate for two complete rotations. Performance was quantified as latency to fall/immobility, with final scores representing the mean duration across three consecutive trials.

Cylinder test. The cylinder test was performed according to established protocols^18^. Following a 30-minute acclimation period in the testing environment, animals were individually placed in an opaque cylindrical testing chamber (height: 30 cm; diameter: 20 cm) with black interior walls and a 2-cm-thick padded floor. Behavioral sessions were initiated immediately upon placement and recorded for 5 min using video monitoring equipment. Forelimb activity was quantified using the following standardized criteria: (1) a valid rearing event required both forelimbs to be elevated above shoulder height and simultaneously contact the cylinder wall; (2) sequential events were recorded only after both forelimbs had fully returned to the chamber floor. Only bilateral forelimb contacts were quantified; separate left or right forelimb contacts were not analyzed. This standardized approach allowed for objective measurement of vertical exploratory behavior while minimizing potential confounding factors related to testing conditions.

Forelimb Akinesia Test (FAS). The forelimb akinesia test was conducted following established methodology^19^. Briefly, the examiner stabilized the animal by gently supporting the hindlimbs with one hand while immobilizing the right forelimb with the other, allowing the left forelimb to maintain contact with the testing surface. Animals were positioned at a 45° angle relative to the horizontal plane and moved laterally along the table edge at a constant velocity (90 cm/10 s). This procedure was repeated for six consecutive trials with 10-second inter-trial intervals. An independent observer, blinded to experimental groups and treatments, quantified forelimb stepping activity during each trial. The final score represented the mean step count across all six trials, ensuring reliable assessment of motor function.

### Immunohistochemistry (IHC)

Animals selected for histological evaluation underwent comprehensive motor function assessment prior to tissue processing. Brain sections were prepared using a vibratome to obtain 30 μm coronal free-floating sections. For dopaminergic marker analysis, sections were immunostained with an anti-tyrosine hydroxylase (TH) primary antibody followed by diaminobenzidine (DAB) peroxidase visualization. Stained sections were imaged using a brightfield microscope (Nikon ECLIPSE50i, Nikon, Tokyo, Japan).

### Statistical analyses

All statistical analyses were performed using R software (version 3.6.3). Quantitative data are presented as mean ± standard deviation (SD) or mean ± standard error of the mean (SEM) as specified in the figure legends. Normality was assessed using the Shapiro-Wilk test. For comparisons involving two factors (rotenone treatment and pharmacological intervention), two-way analysis of variance (ANOVA) was applied, followed by post-hoc tests (Tukey’s or Bonferroni’s) for multiple comparisons. For single-factor comparisons, either Student’s t-test (for normally distributed data) or Mann-Whitney U test (for non-normally distributed data) was used. Outlier analysis was performed using Grubbs’ test (α = 0.05); no extreme outliers were identified, and no data points were excluded. Statistical significance was defined as *P* < 0.05, with significance levels denoted as ^*^P < 0.05, ^**^P < 0.01, and ^***^P < 0.001.

## Results

### Cytotoxic effects of rotenone in PC12 cells

**Fig. 1:**
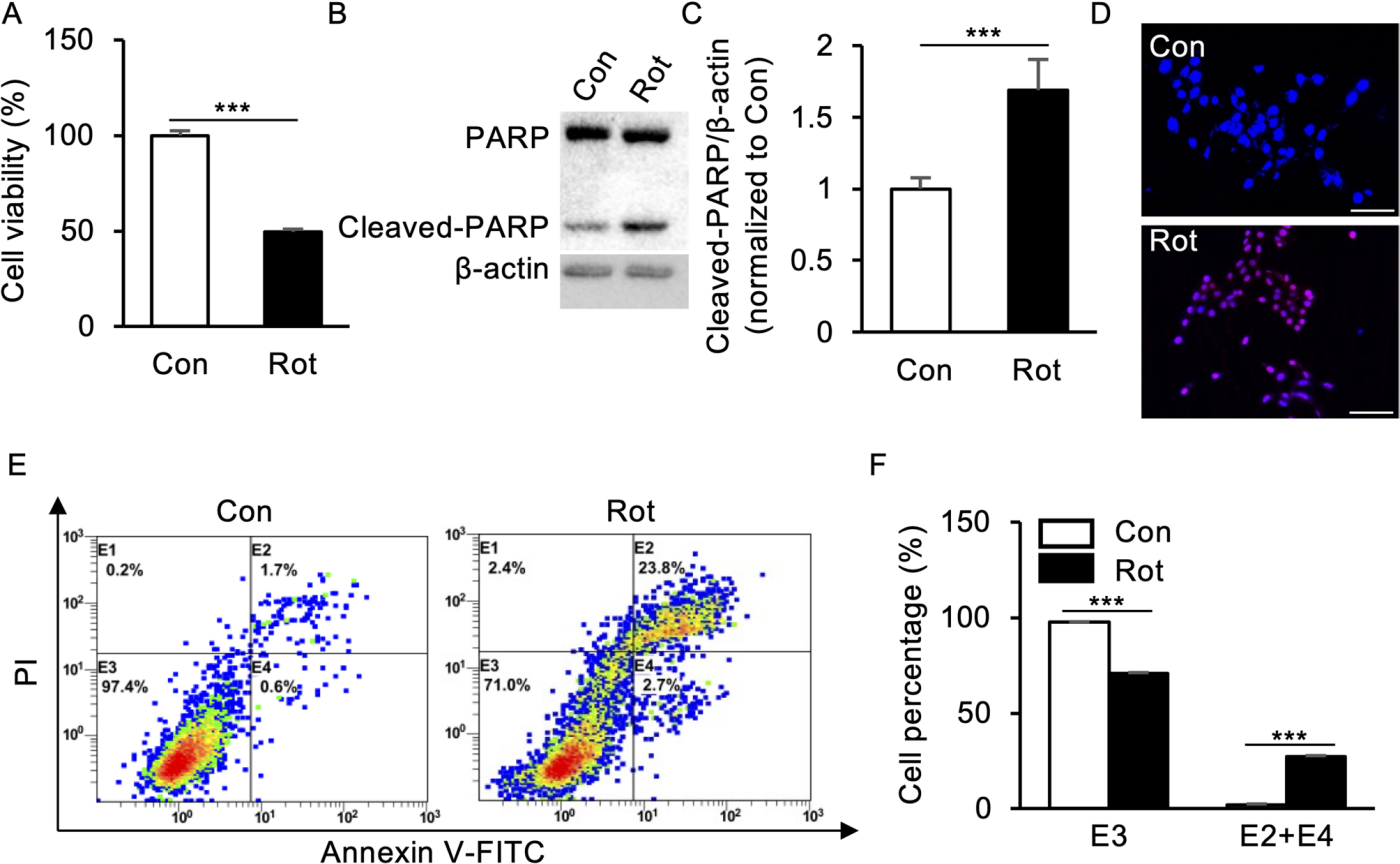
Rotenone induces cytotoxic effects in PC12 cells. (**A**) Cell viability assessed by SRB assay following 24 h treatment with 200 nM rotenone (Rot). (**B**) Western blot analysis of cleaved-PARP expression. (Cropped blot images are shown; see Supplementary Fig. 3 for full immunoblots). (**C**) Quantification of cleaved-PARP/β-actin ratio normalized to control (Con). (**D**) TUNEL-positive cells are shown in red (scale bar = 50 μm); nuclei are counterstained with DAPI (blue). (**E**) Flow cytometry plots of apoptosis using annexin V-FITC/PI staining. (**F**) Quantitative analysis of viable (PI⁻ annexin V⁻, E3) and apoptotic (PI⁺ annexin V⁺, E2; and PI⁻ annexin V⁺, E4) cell populations. Data represent mean ± SD from five independent experiments. ***P < 0.001.

To establish an experimental paradigm, we first performed dose‑response studies with rotenone (Rot) in PC12 cells. A concentration showing intermediate cytotoxicity was selected for subsequent experiments (Supplementary Fig. 1 and Fig. 1A). Western blot analysis showed an increase in cleaved‑PARP levels following Rot treatment compared with control conditions (Fig. 1B,C). TUNEL staining revealed a higher proportion of TUNEL‑positive cells upon Rot exposure, suggesting enhanced DNA fragmentation (Fig. 1D). Flow cytometric analysis further indicated an increased percentage of apoptotic cells in Rot‑treated groups (Fig. 1E,F). Together, these observations are consistent with the interpretation that Rot triggers apoptotic cell death in this neuronal model.

### Rotenone‑induced neuronal apoptosis is associated with glycolysis‑dependent MG‑Hs formation

**Fig. 2:**
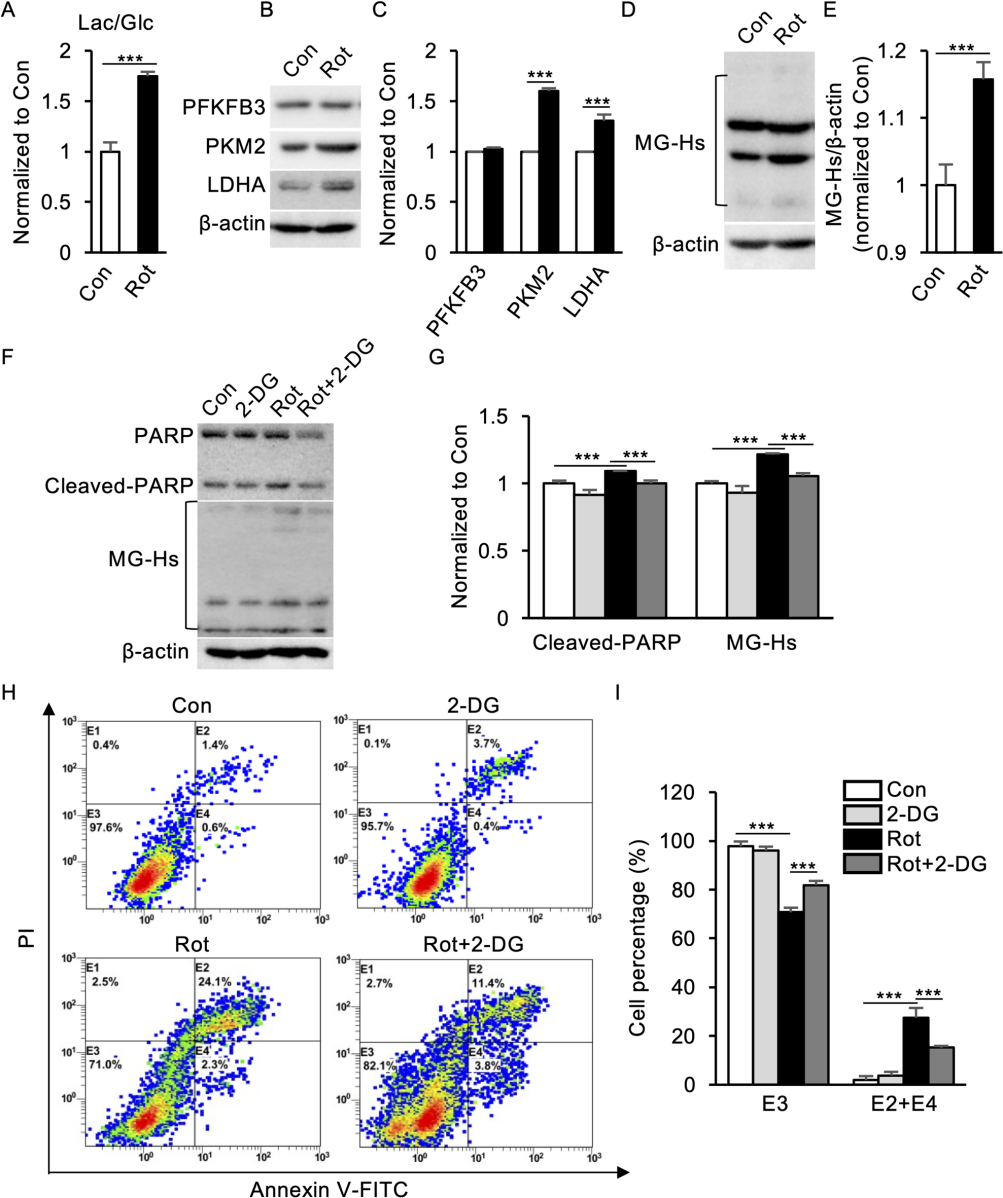
Rotenone induces neuronal apoptosis through glycolytic activation in PC12 cells. (**A**) Lactate-to-glucose consumption ratio following 24 h rotenone (Rot, 200 nM) treatment. (**B**) Western blot analysis of glycolytic enzymes (PFKFB3, PKM2, LDHA) with β-actin loading control. (Cropped blot images are shown; see Supplementary Fig. 4A for full immunoblots) (**C**) Densitometric quantification of glycolytic enzyme expression normalized to β-actin. (**D**) MG-Hs protein adducts detected by immunoblotting. (Cropped blot images are shown; see Supplementary Fig. 4B for full immunoblots). (**E**) Quantification of MG-Hs levels normalized to β-actin. (**F**) Cleaved-PARP and MG-Hs expression in cells pretreated with 2-DG (25 µM, 2 h) prior to Rot exposure. (Cropped blot images are shown; see Supplementary Fig. 4C for full immunoblots). (**G**) Quantification of cleaved-PARP and MG-Hs levels. (**H**) Flow cytometry profiles of apoptotic cells with/without 2-DG treatment. (**I**) Quantitative analysis of viable (PI^−^Annexin V^−^) and apoptotic (PI^+^Annexin V^+^ and PI^−^Annexin V^+^) populations. Data represent mean ± SD of five independent experiments. ^***^P < 0.001.

Rotenone (Rot), a classical mitochondrial complex I inhibitor, has been extensively characterized for its toxicity through disruption of oxidative phosphorylation^20–22^. However, emerging evidence challenges this canonical view, demonstrating complex I-independent mechanisms of Rot-induced cytotoxicity^11^. Our current findings reveal a previously unrecognized metabolic effect of Rot, showing significant activation of glycolytic flux in PC12 cells. This was evidenced by both increased lactate production relative to glucose consumption and upregulation of key glycolytic enzymes (Fig. 2A and C). These observations suggest a compensatory metabolic shift, wherein Rot-induced impairment of mitochondrial ATP production triggers enhanced glycolytic activity to maintain cellular energy homeostasis. This metabolic reprogramming represents a potentially critical adaptive response to mitochondrial dysfunction in neuronal cells.

The mechanistic link between rotenone-induced glycolytic activation and its cytotoxic effects was further investigated through analysis of methylglyoxal (MG) metabolism. As a reactive glycolytic byproduct, MG undergoes non-enzymatic modification with arginine residues to form methylglyoxal-derived hydroimidazolones (MG-Hs), known mediators of apoptotic cell death^23–25^. Our data demonstrate significant accumulation of MG-Hs in rotenone-treated PC12 cells (Fig. 2D and E), suggesting a potential pathway for glycolytic-mediated cytotoxicity.

To establish causality, we employed the glycolytic inhibitor 2-deoxyglucose (2-DG) at concentrations previously shown to be well-tolerated^26^. This intervention effectively attenuated both MG-Hs formation and apoptotic markers (cleaved-PARP) in rotenone-exposed cells (Fig. 2F and G). Complementary flow cytometric analysis using Annexin V-FITC/PI staining confirmed the reduction in apoptotic cell populations following 2-DG treatment (Fig. 2H and I). These consistent findings support a model wherein rotenone promotes neuronal apoptosis through glycolysis-dependent accumulation of cytotoxic MG-Hs adducts.

**Fig. 3:**
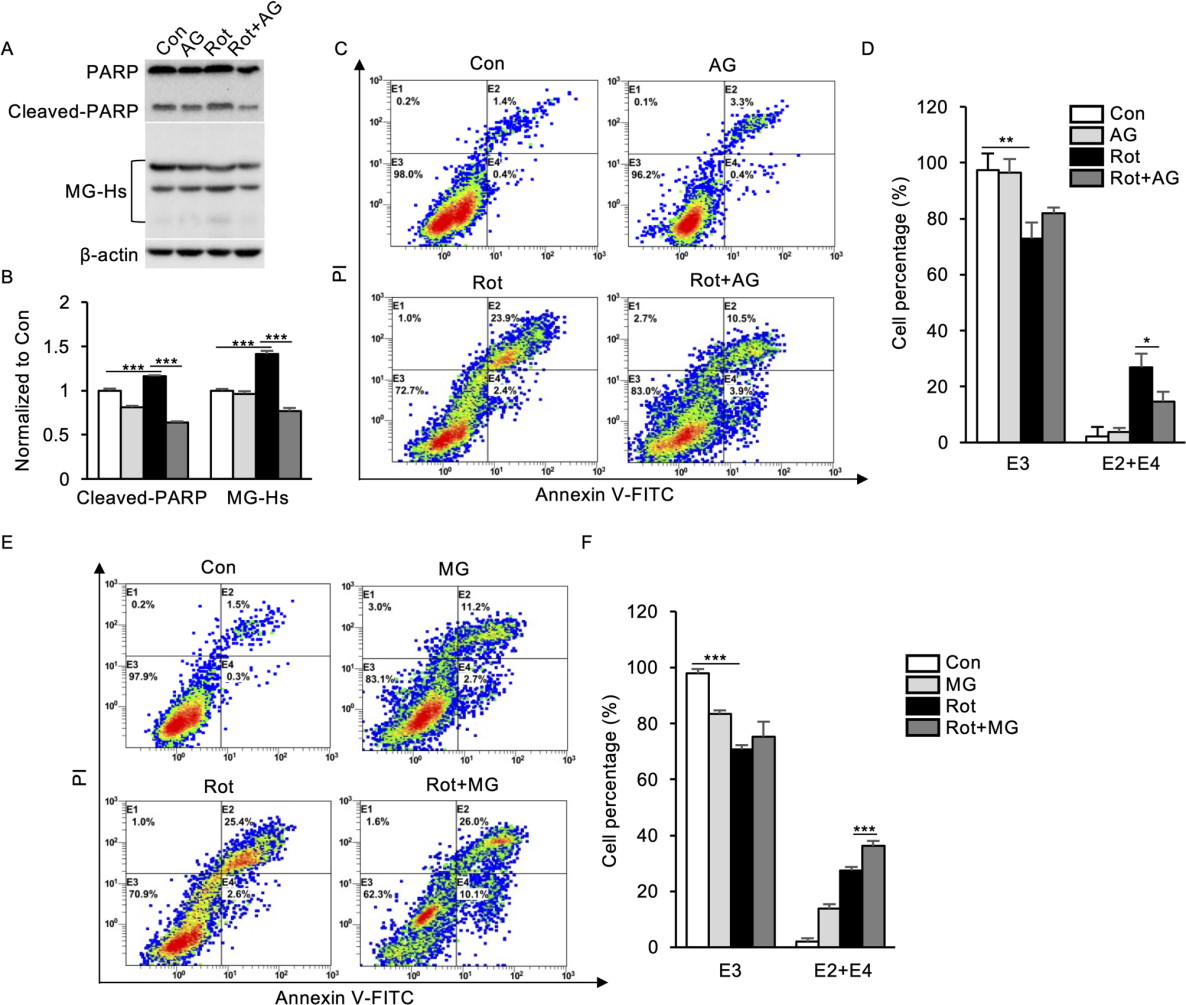
MG-Hs-dependent apoptotic pathway in rotenone-treated PC12 cells. (**A**) Western blot analysis of cleaved-PARP and MG-Hs expression following pretreatment with the methylglyoxal scavenger aminoguanidine (AG, 100 µM, 2 h) and subsequent rotenone (Rot, 200 nM, 24 h) treatment. (Cropped blot images are shown; see Supplementary Fig. 4D for full immunoblots). (**B**) Quantification of cleaved-PARP/β-actin and MG-Hs/β-actin ratios normalized to control (Con). (**C**) Flow cytometry profiles of apoptotic cells treated with AG or vehicle prior to Rot exposure. (**D**) Quantitative analysis of viable (PI^−^Annexin V^−^) and apoptotic (PI^+^Annexin V^+^ and PI^−^Annexin V^+^) populations. (**E**) Flow cytometry analysis of cells pretreated with exogenous methylglyoxal (MG, 25 µM, 2 h) before Rot administration. (**F**) Quantification of viable and apoptotic cell populations following MG + Rot treatment. Data represent mean ± SD of five biologically independent experiments. ***P < 0.001.

To establish the causal role of methylglyoxal-derived hydroimidazolones (MG-Hs) in rotenone-induced apoptosis, we employed aminoguanidine (AG), a selective MG scavenger^27^. AG treatment effectively prevented both MG-Hs accumulation and apoptotic activation in rotenone-exposed PC12 cells. Western blot analysis demonstrated significant reduction in cleaved-PARP and MG-Hs levels following AG intervention (Fig. 3A,B). Flow cytometric assessment confirmed the anti-apoptotic effect of AG, showing marked decrease in apoptotic cell populations (Fig. 3C,D). Complementary experiments with exogenous methylglyoxal (MG) treatment exacerbated apoptotic responses in rotenone-treated cells (Fig. 3E,F), providing additional evidence for MG-Hs-mediated cytotoxicity. Collectively, these findings demonstrate that rotenone triggers neuronal apoptosis primarily through glycolysis-dependent MG-Hs formation, establishing a clear metabolic pathway linking glycolytic activation to cell death execution.

### Shikonin attenuates rotenone-induced neurotoxicity via PKM2-dependent metabolic regulation

**Fig. 4:**
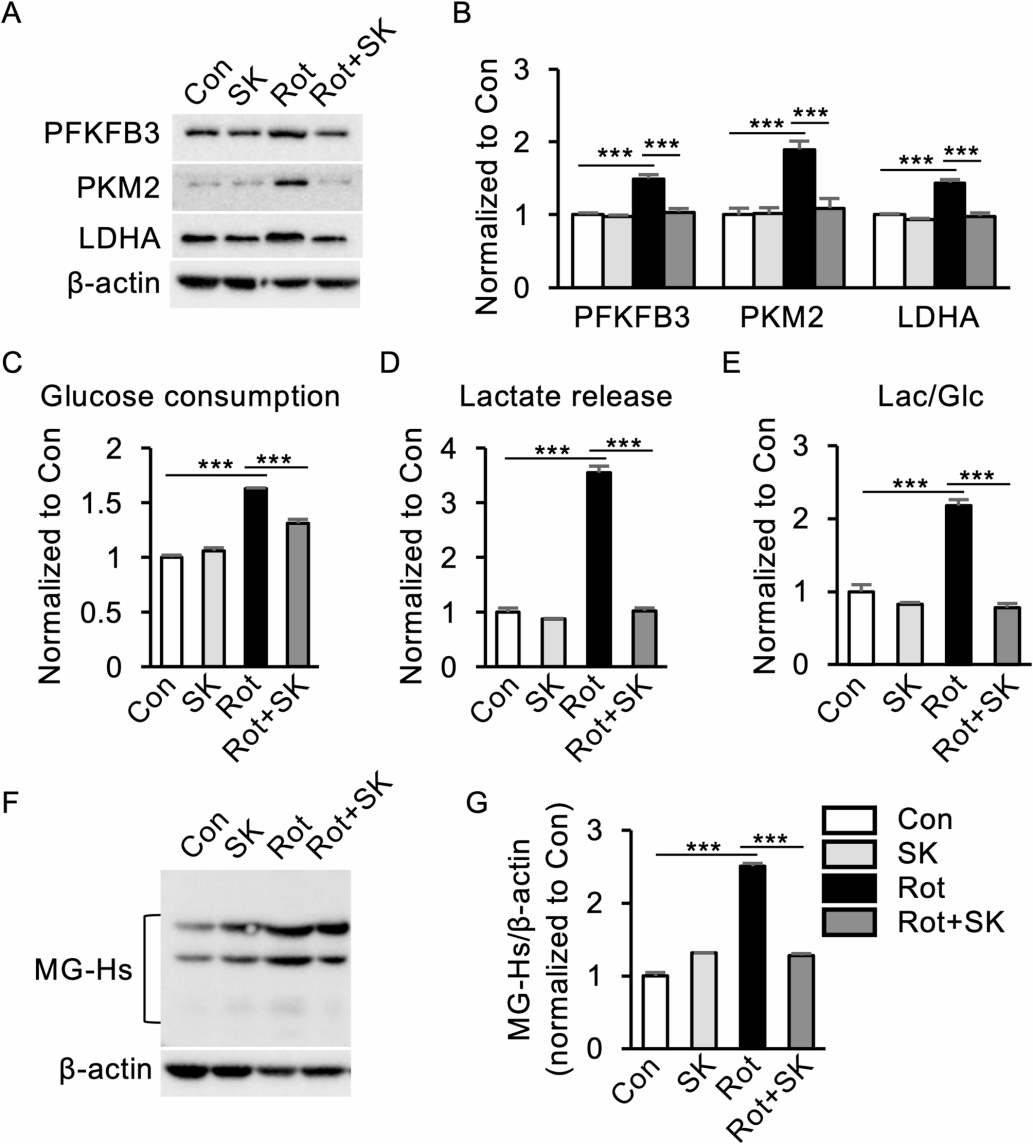
Shikonin attenuates rotenone-induced glycolytic activation and MG-Hs formation in PC12 cells. (**A**) Western blot analysis of glycolytic enzymes (PFKFB3, PKM2, LDHA) following pretreatment with shikonin (SK, 50 nM, 2 h) and subsequent co‑treatment with rotenone (Rot, 200 nM, 24 h). (Cropped blot images are shown; see Supplementary Fig. 5A for full immunoblots) (**B**) Quantification of glycolytic enzyme expression normalized to β-actin. (**C**) Glucose consumption, (**D**) lactate release, and (**E**) lactate-to-glucose ratio (Lac/Glc) measured by colorimetric assays. (**F**) MG-Hs protein adducts detected by immunoblotting. (Cropped blot images are shown; see Supplementary Fig. 5B for full immunoblots). (**G**) Quantification of MG-Hs levels normalized to β-actin. Data represent mean ± SD of five independent experiments. ***P < 0.001.

Building on our findings that glycolytic activation mediates rotenone (Rot) cytotoxicity, we investigated shikonin, a natural naphthoquinone derived from *Lithospermum erythrorhizon* (Boraginaceae), as a potential therapeutic agent. As a known PKM2 inhibitor with established pharmacological properties^28^, shikonin effectively attenuated Rot-induced metabolic dysregulation in PC12 cells. Our results demonstrate shikonin’s capacity to suppress glycolytic hyperactivity, as evidenced by: (i) Downregulation of key glycolytic enzymes (PFKFB3, PKM2, and LDHA; Fig. 4A,B); (ii) Reduction in glucose consumption and lactate production (Fig. 4C,E); (iii) Decreased MG-Hs accumulation (Fig. 4F,G).

**Fig. 5:**
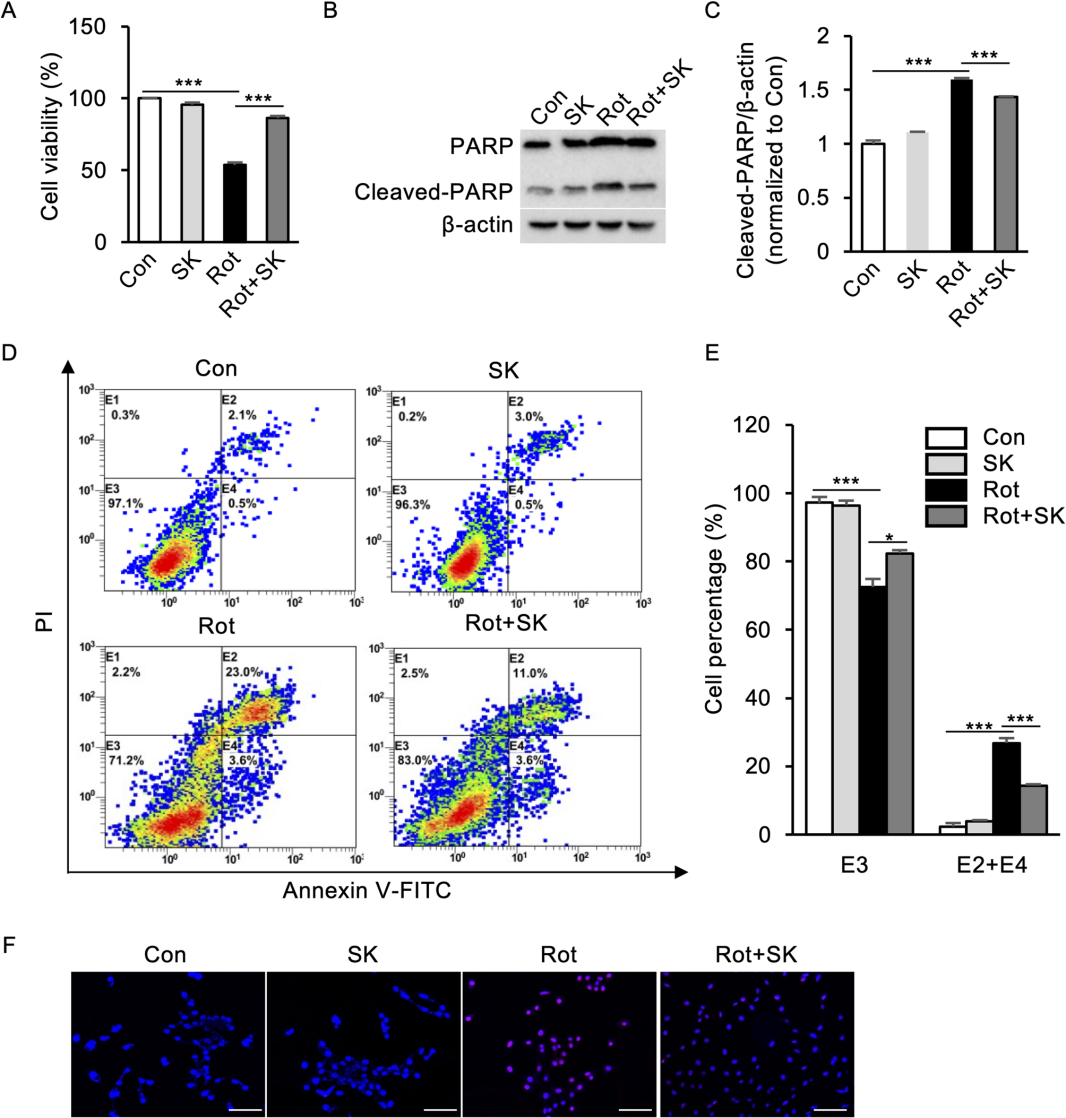
Shikonin attenuates rotenone-induced apoptosis in PC12 cells through glycolytic inhibition. (**A**) Cell viability assessed by SRB assay following treatment with shikonin (SK, 50 nM, 2 h pretreatment) and subsequent co‑treatment with rotenone (Rot, 200 nM, 24 h). (**B**) Western blot analysis of cleaved-PARP expression with β-actin loading control. (Cropped blot images are shown; see Supplementary Fig. 6 for full immunoblots) (**C**) Quantification of cleaved-PARP/β-actin ratio normalized to control (Con). (**D**) Flow cytometry analysis of apoptotic cells. (**E**) Quantitative assessment of viable (PI^−^Annexin V^−^) and apoptotic (PI^+^Annexin V^+^ and PI^−^Annexin V^+^) populations. (**F**) TUNEL staining (red, scale bar = 50 μm). Data represent mean ± SD of five biologically independent experiments. ^***^P < 0.001.

To evaluate the neuroprotective potential of shikonin, we systematically examined its effects on rotenone (Rot)-induced apoptosis in PC12 cells. As shown in Fig. 5A, shikonin significantly attenuated Rot-induced cytotoxicity in cell viability assays. Western blot analysis further demonstrated that shikonin treatment markedly reduced the expression of cleaved-PARP, a key mediator of apoptosis (Fig. 5B, C). Consistent with these findings, flow cytometric analysis confirmed a decrease in the proportion of apoptotic cells upon shikonin exposure (Fig. 5D, E). Additional support was provided by TUNEL staining, which revealed a reduction in TUNEL-positive neurons following shikonin treatment, indicating diminished DNA fragmentation (Fig. 5F). Collectively, these results from multiple experimental approaches substantiate the neuroprotective effect of shikonin against Rot-induced neuronal apoptosis.

To investigate whether the neuroprotective effects of shikonin involve PKM2 inhibition, we employed genetic approaches to modulate PKM2 expression. In Rot-treated cells, TUNEL staining revealed a pronounced level of apoptosis under normal PKM2 expression conditions (Supplementary Fig. 2B). In contrast, PKM2 knockdown significantly attenuated Rot-induced neuronal damage (Supplementary Fig. 2B). Furthermore, overexpression of PKM2 partially reversed the protective effect of shikonin against Rot-induced neurotoxicity (Supplementary Fig. 2D). Collectively, these genetic manipulations demonstrate that PKM2 inhibition mitigates Rot-induced neuronal apoptosis and that shikonin exerts its neuroprotective role, at least in part, through PKM2.

Based on these and preceding findings, we propose a mechanistic model underlying shikonin-mediated neuroprotection: (i) Rotenone triggers neuronal apoptosis by enhancing glycolysis and promoting methylglyoxal-hydroimidazolone (MG-Hs) accumulation; (ii) Shikonin counteracts this process by targeting the glycolytic pathway; (iii) PKM2 serves as a critical regulatory node, whose inhibition alleviates metabolic stress-induced neuronal injury.

### Therapeutic effects of Shikonin in rotenone-induced Parkinson’s disease: behavioral and neuroanatomical recovery

**Fig. 6:**
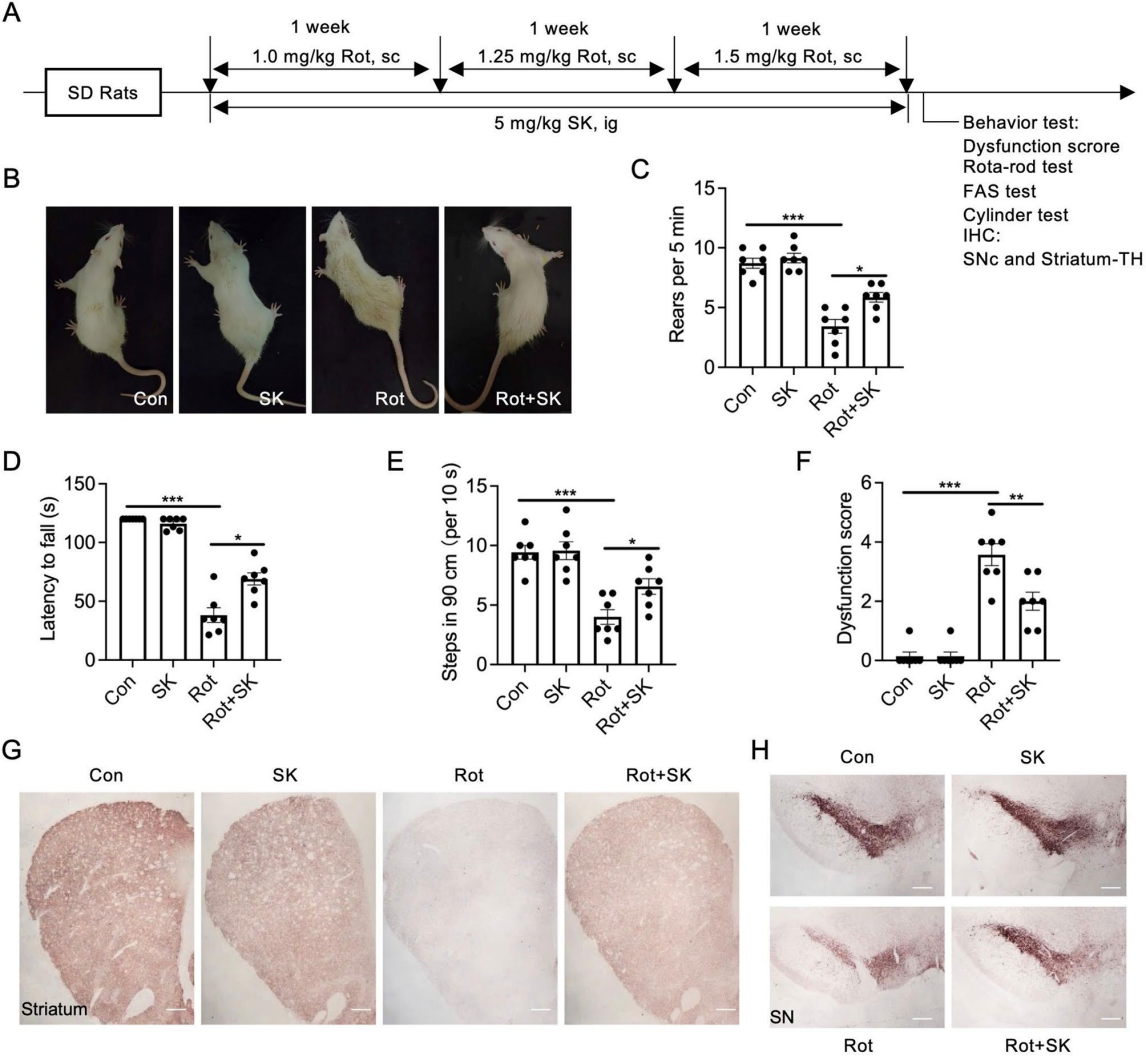
Shikonin ameliorates motor deficits and neuropathology in rotenone-induced Parkinsonian rats. (**A**) Experimental timeline of rotenone (Rot) induction and shikonin (SK) treatment. (**B**) Representative photographs of rats from each treatment group (Con, SK, Rot, and Rot + SK), illustrating the overall behavioral phenotype at the experimental endpoint, including posture, limb position, and spontaneous activity level. (**C**) Quantitative assessment of motor dysfunction scores (*n* = 7 per group). (**D**–**F**) Motor performance evaluated by rotarod (**D**), forelimb akinesia (FAS) (**E**), and cylinder (**F**) tests (*n* = 7). (**G**) Tyrosine hydroxylase (TH) immunostaining of striatal terminals. (**H**) TH+ neuronal survival in substantia nigra pars compacta (SNpc). Scale bars: 500 μm (striatum), 1000 μm (SNpc). Data represent mean ± SEM. ***P < 0.001.

To evaluate the disease-modifying potential of shikonin, we conducted a longitudinal intervention study in rotenone (Rot)-induced parkinsonian rats (Fig. 6A). Three weeks of daily oral shikonin administration produced robust therapeutic effects across multiple outcome measures.

Behavioral assessments revealed shikonin-mediated improvements in: (i) composite dysfunction scores (Fig. 6B, C), (ii) motor coordination (rotarod test), (iii) forelimb akinesia (FAS test), and (iv) exploratory behavior (cylinder test) (Fig. 6D–F). Complementary immunohistochemical analysis demonstrated substantial degeneration of nigrostriatal dopaminergic integrity, with shikonin treatment attenuating both striatal terminal fiber loss (Fig. 6G) and substantia nigra pars compacta (SNc) neuronal degeneration (Fig. 6H). These in vivo improvements are consistent with the protective mechanisms observed in vitro, and future studies may further explore the molecular correlates of shikonin’s actions, including its effects on PKM2, glycolysis, and MG-Hs in brain tissue, as well as its well-documented anti-inflammatory and anti-oxidant properties.

Together, these coordinated functional and neuroanatomical improvements establish shikonin’s capacity to mitigate disease progression in this preclinical PD model. The concordance between behavioral recovery and dopaminergic pathway preservation underscores the translational potential of shikonin as a promising neuroprotective agent targeting Parkinson’s disease pathogenesis.

## Discussion

Rotenone is widely used to model Parkinson’s disease (PD) because it recapitulates key features such as α‑synuclein aggregation, mitochondrial dysfunction, and oxidative stress^29–37^. Although these effects have traditionally been linked to complex I inhibition, evidence from Ndufs4‑deficient mice indicates that rotenone can induce dopaminergic neuron death even in the absence of a functional complex I^11^, suggesting that additional mechanisms contribute to its neurotoxicity.

The present study provides evidence consistent with the involvement of glycolytic activation in rotenone-induced neuronal damage. Our in vitro data show that rotenone increases glycolytic flux, leading to accumulation of methylglyoxal (MG)-derived hydroimidazolones (MG-Hs)^38–40^. This observation is supported by the following findings: (i) the glycolytic inhibitor 2-deoxyglucose (2-DG) reduced both MG-Hs and apoptosis; (ii) the MG scavenger aminoguanidine (AG) attenuated apoptosis; and (iii) exogenous MG exacerbated cell death. Together, these results are consistent with a model in which rotenone-induced glycolysis generates excess MG, which then forms cytotoxic MG-Hs adducts and promotes apoptosis. Under physiological conditions, MG is primarily detoxified by the glyoxalase system (Glo1/Glo2)^41^. Although we did not directly assess whether rotenone affects glyoxalase expression or activity, nor whether shikonin might modulate this pathway, MG-Hs accumulation in our experiments could result from increased MG production, impaired detoxification, or both. Future studies should therefore examine the contribution of the glyoxalase system to rotenone neurotoxicity and whether it represents an additional point of therapeutic intervention.

Our data do not resolve the precise temporal sequence; the observed glycolytic upregulation is likely secondary to complex I inhibition. Importantly, the protective effect of shikonin demonstrates that this downstream glycolytic branch is a meaningful contributor to cell death, even if it is not the initiating event. We also identified shikonin as a compound that effectively reduces rotenone-induced apoptosis in vitro, and it is known to inhibit PKM2^42–46^. To directly assess whether PKM2 mediates the observed protective effects, we performed genetic modulation experiments: PKM2 knockdown using siRNA significantly attenuated rotenone-induced neuronal apoptosis, while PKM2 overexpression partially reversed the protective effect of shikonin, demonstrating that PKM2 plays a critical role in mediating both rotenone-induced neuronal apoptosis and the neuroprotective action of shikonin. Although shikonin has a well-documented multi-target pharmacological profile (including effects on NF-κB, STAT3, kinases, and anti-inflammatory/anti-oxidant activities)^46^, our genetic evidence establishes PKM2 as a key functional target in the context of rotenone-induced glycolytic dysregulation. In a rotenone-induced rat model, oral administration of shikonin robustly improved motor function and preserved nigrostriatal dopaminergic neurons, consistent with the in vitro defined PKM2–glycolysis–MG-Hs axis. Future studies may further examine the molecular correlates of shikonin’s actions in brain tissue, including PKM2 activity and MG-Hs levels; nonetheless, the convergence of in vitro genetic evidence and in vivo functional improvement strongly supports PKM2 as a mediator of shikonin’s neuroprotective effects in Parkinson’s disease models.

Taken together, our findings identify a previously unrecognized pathway downstream of complex I inhibition—involving glycolytic activation and MG-Hs accumulation—that contributes to rotenone neurotoxicity. Our data also suggest that shikonin counteracts this process, at least in part through PKM2 inhibition. However, we acknowledge that much of the evidence is associative in nature, that shikonin may have additional targets, and that direct in vivo molecular validation is still lacking. Therefore, the proposed pathway should be regarded as a hypothesis-generating model rather than a definitively established mechanism. Independent replication and more definitive causal experiments—such as tissue-specific PKM2 knockout or rescue of MG-Hs accumulation following PKM2 inhibition—are necessary to further substantiate this cascade and to assess its translational relevance for Parkinson’s disease.

## Conclusion

This study presents evidence consistent with the involvement of glycolytic activation and methylglyoxal‑derived hydroimidazolones (MG‑Hs) accumulation in rotenone‑induced neurotoxicity. Our in vitro data suggest that rotenone increases glycolytic flux, leading to MG‑Hs formation and subsequent apoptosis. The natural compound shikonin attenuates these effects in cell culture, with PKM2 appearing to be one of the contributing targets. In a rotenone‑treated rat model, shikonin improves motor function and preserves nigrostriatal dopaminergic neurons, supporting the translational relevance of targeting glycolytic dysregulation in Parkinson’s disease.

Nevertheless, several limitations should be acknowledged. First, our in vitro experiments were conducted exclusively in the PC12 cell line; primary midbrain dopaminergic neurons or human iPSC‑derived neurons would provide greater translational confidence. Second, the rotenone model, while useful, does not fully recapitulate idiopathic PD features such as α‑synuclein pathology; confirmation in other models (e.g., α‑synuclein transgenic or 6‑OHDA) is needed. Third, only male rats were used, leaving potential sex‑dependent effects unexplored. Fourth, shikonin is known to have multi‑target pharmacology; although our genetic evidence supports PKM2 as a key mediator, off‑target contributions cannot be excluded. Finally, we did not directly measure PKM2 activity or MG‑Hs levels in brain tissue, which would strengthen the mechanistic link.

Future studies should address these limitations by employing tissue‑specific PKM2 modulation (e.g., conditional knockout or viral‑mediated knockdown in the substantia nigra), directly quantifying glycolytic markers and MG‑Hs in brain tissue, testing both sexes, and evaluating shikonin in additional PD models. Such efforts will further elucidate the precise molecular cascade underlying shikonin’s neuroprotective actions and assess its disease‑modifying potential.

## Supplementary Information

Below is the link to the electronic supplementary material.

Supplementary Material 1

## Data Availability

The date and materials are available from the cor-responding author upon reasonable request.
