# Supplementary material for: PKM2-driven glycolysis mediates rotenone neurotoxicity via MG-Hs in Parkinson’s disease
Source: Sci Rep. 2026 May 30;16:24690. doi: 10.1038/s41598-026-54865-7 (PMC13454143; doi:10.1038/s41598-026-54865-7)
Supplement: Supplementary file 1 [file 41598_2026_54865_MOESM1_ESM.docx]

**PKM2-driven glycolysis mediates rotenone neurotoxicity via MG-Hs in Parkinson's disease**

Supplementary materials include:

Supplementary Figures 1 to 7

**Supplementary Figure 1. Concentration-dependent effects of rotenone on PC12 cell viability.** Data represent mean ± SD of five biologically independent experiments. ^*^*P* < 0.05, ^***^*P* < 0.001 versus Con group.

**Supplementary Figure 2. The role of PKM2 in rotenone (Rot)-induced neuronal apoptosis in PC12 cells.** (A) Immunoblot analysis validating the efficiency of PKM2 knockdown. (Cropped blot images are shown; see Supplementary Figure 7A for full immunoblots) (B) Representative TUNEL staining images showing the effect of PKM2 knockdown on Rot-induced neuronal apoptosis. Scale bar = 50 μm. (C) Immunoblot analysis confirming PKM2 overexpression. (Cropped blot images are shown; see Supplementary Figure 7B for full immunoblots) (D) TUNEL staining demonstrating that PKM2 overexpression (OE-PKM2) attenuates the neuroprotective effect of shikonin (SK) against Rot-induced apoptosis.

**Supplementary Figure 3.** Uncropped figures from western blots. Uncropped western blot images that correspond to Figure 1B.

**Supplementary Figure 4. Uncropped figures from western blots.** Uncropped western blot images that correspond to Figure 2B (A), Figure 2D (B), Figure 2F (C), Figure 3A (D).

**Supplementary Figure 5. Uncropped figures from western blots.** Uncropped western blot images that correspond to Figure 4A (A), Figure 4F (B).

**Supplementary Figure 6.** **Uncropped figures from western blots.** Uncropped western blot images that correspond to Figure 5B.

**Supplementary Figure 7.** **Uncropped figures from western blots.** Uncropped western blot images that correspond to Supplementary Figure 2A (A), Supplementary Figure 2C (B).
